# Genetic insights into the association of statin and newer nonstatin drug target genes with human longevity: a Mendelian randomization analysis

**DOI:** 10.1186/s12944-023-01983-0

**Published:** 2023-12-12

**Authors:** Han Chen, Xiaoying Zhou, Jingwen Hu, Shuo Li, Zi Wang, Tong Zhu, Hong Cheng, Guoxin Zhang

**Affiliations:** 1 Department of Gastroenterology, The First Affiliated Hospital of Nanjing Medical University, 300# Guangzhou Road, Nanjing, 210029 People’s Republic of China; 2 Department of Cardiology, The First Affiliated Hospital of Nanjing Medical University, Nanjing, People’s Republic of China; 3 Department of Neurology, The First Affiliated Hospital of Nanjing Medical University, Nanjing, People’s Republic of China; 4 Branch of Health Promotion and Education, Jiangsu Anti-aging Association, Nanjing, People’s Republic of China

**Keywords:** Drug-target Mendelian randomization, *CETP*, *APOC3*, *LDLR*, Human longevity

## Abstract

**Background:**

It remains controversial whether the long-term use of statins or newer nonstatin drugs has a positive effect on human longevity. Therefore, this study aimed to investigate the genetic associations between different lipid-lowering therapeutic gene targets and human longevity.

**Methods:**

Two-sample Mendelian randomization analyses were conducted. The exposures comprised genetic variants that proxy nine drug target genes mimicking lipid-lowering effects *(LDLR, HMGCR, PCKS9, NPC1L1, APOB, CETP, LPL, APOC3,* and *ANGPTL3*). Two large-scale genome-wide association study (GWAS) summary datasets of human lifespan, including up to 500,193 European individuals, were used as outcomes. The inverse-variance weighting method was applied as the main approach. Sensitivity tests were conducted to evaluate the robustness, heterogeneity, and pleiotropy of the results. Causal effects were further validated using expression quantitative trait locus (eQTL) data.

**Results:**

Genetically proxied *LDLR* variants, which mimic the effects of lowering low-density lipoprotein cholesterol (LDL-C), were associated with extended lifespan. This association was replicated in the validation set and was further confirmed in the eQTL summary data of blood and liver tissues. Mediation analysis revealed that the genetic mimicry of *LDLR* enhancement extended lifespan by reducing the risk of major coronary heart disease, accounting for 22.8% of the mediation effect. The genetically proxied *CETP* and *APOC3* inhibitions also showed causal effects on increased life expectancy in both outcome datasets. The lipid-lowering variants of *HMGCR, PCKS9, LPL,* and *APOB* were associated with longer lifespans but did not causally increase extreme longevity. No statistical evidence was detected to support an association between *NPC1L1* and lifespan.

**Conclusion:**

This study suggests that *LDLR* is a promising genetic target for human longevity. Lipid-related gene targets, such as *PCSK9, CETP*, and *APOC*3, might potentially regulate human lifespan, thus offering promising prospects for developing newer nonstatin therapies.

**Supplementary Information:**

The online version contains supplementary material available at 10.1186/s12944-023-01983-0.

## Background

Human longevity is a remarkably complex phenotype modulated by both genetic and environmental determinants. Epidemiological studies have shown that environmental elements play a significant role in determining lifespan [1, 2]. In recent years, the study of ageing and the processes that limit lifespan has gained significant scientific credibility due to the discovery of gene variants that have been found to increase the lifespan of multicellular model organisms [3]. With the emergence of large-scale genome-wide association study (GWAS), several genetic variants have also been revealed to be significantly associated with life expectancy. Genetic biomarkers, such as *APOE* and *FOXO3*, have been frequently reported to be associated with longevity [4–6]. In addition to the previously identified age-related genes, numerous associations involving other genes have been identified by different studies, providing new perspectives for detecting novel genetic pathways associated with lifespan. *Timmers* et al. [7] conducted a large-scale longevity-associated GWAS analysis with 1 million European participants and revealed several newer candidate biomarkers for longevity, including the low-density lipoprotein receptor (*LDLR*) and lipoprotein lipase (*LPL*). Interestingly, *LDLR* and *LPL* are two cardioprotective variants and are the key modulators of lipid-lowering drugs targeting low-density lipoprotein cholesterol (LDL-C) and triglycerides [8]. This finding suggests that there may be a genetic association between lipid-lowering agents and life expectancy.

In recent years, the relationships of circulating lipids and blood lipid-related phenotypes with human lifespan have been explored through Mendelian randomization (MR) analyses [9]. Ni et al. investigated the impact of modifiable lifestyles on longevity and found that an increase in BMI can causally reduce lifespan by increasing serum lipid levels [10]. Similarly, Daghlas et al. conducted an MR study that showed a significant association between high serum lipid levels and shortened lifespans [11]. These findings indicate that lipid-lowering therapies have a positive impact on overall lifespan.

Statins are a commonly used drugs that target the 3-hydroxy-3-methylglutaryl coenzyme A reductase (*HMGCR*) to lower circulating LDL-C levels. Studies have shown that statin use can reduce the risk of all-cause mortality [12] and increase lifespan [13]. However, the effects of nonstatin lipid-lowering therapies such as ezetimibe and evolocumab on lifespan are still unclear. Additionally, there is limited research on the genetic association between various lipid-lowering drugs and human lifespan. Therefore, this study aimed to investigate the genetic impact of different lipid-lowering drugs on human longevity by examining gene targets.

Mendelian randomization (MR) is a relatively new approach that has been utilized in recent publications to examine the potential connections between genetically regulated biomarkers or aging-related pathways and human lifespan [7, 14–17]. The analysis of drug-targets using MR is a component of MR investigations, however, only genetic variations within drug target genes or in their immediate vicinity are preserved [18]. The effect of genetic variation within genes encoding drug targets could explain possible causal relationships of drug target manipulation with modulation of exposure and outcome. Therefore, this study uses MR as a valuable tool to test whether there is a causal effect of different lipid-reducing drugs on human lifespan. The present research will provide genetic insights into the potential advantages of prolonged usage of statins and novel nonstatin medications.

## Methods

### Study design and data sources

**Fig. 1:**
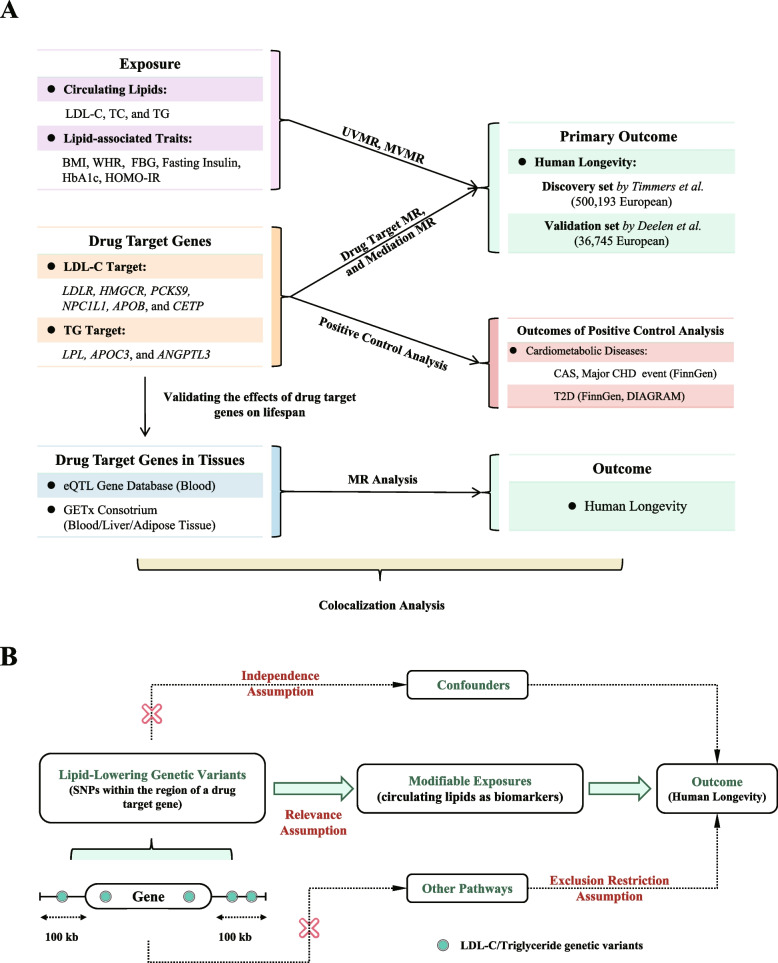
Flowchart of the study design and MR assumptions. **a** Outline of the study design; **b** Assumptions of the Mendelian randomization study: in this study, genetic instruments were selected to represent the pharmacological modulation of drug target proteins based on their associations with circulating lipid concentrations (relevance assumption). Additionally, it was assumed that the selected genetic variants are not associated with confounding factors (independence assumption). The third assumption was that genetic variants should not affect human lifespan through other pathways (exclusion restriction assumption)

This study was conducted in accordance with the Guidelines for Strengthening the Reporting of Observational Studies in Epidemiology using Mendelian Randomization (STROBE-MR) [19] (Table S1). The study design is illustrated in Fig. 1A.

This study consisted of three parts. First, a two-sample MR analysis was conducted to explore causal relationships between lipid/lipid-associated traits and human lifespan. The exposure traits comprised circulating lipids, including total cholesterol (TC), total triglycerides (TG) and LDL-C as well as lipid-associated traits such as body mass index (BMI), waist-to-hip ratio (WHR), fasting blood glucose (FBG), HbA1c, fasting insulin (FI), and homeostatic model assessment of insulin resistance (HOMA-IR). Univariate and multivariate MR analyses [20] were both conducted to estimate the causal effects of circulating lipids on human longevity. Two different types of lifespan-associated GWAS data were used as the outcome, including discovery and validation datasets. The discovery dataset comprises the largest GWAS dataset that studies human life expectancy as the continuous outcome in the general population [7]. The validation set contains large-scale GWAS data [21] focusing on the differences in genetic variation between the extreme cases of longevity individuals surviving past a certain age threshold and control individuals (binary outcome). The two GWAS datasets can offer evidence on whether lipid-lowering medications can increase lifespan or significantly enhance the occurrence of extreme longevity or if they apply to both.

Second, a drug-target MR analysis was conducted to explore genetic associations between lipid-lowering drug-target genes and longevity. The classification of lipid-lowering drugs and their target genes was based on the latest expert consensus and guidelines regarding lipid-lowering therapies [22, 23], which are summarized in Table 1. Genetic variants of genes that encode lipid-lowering drug target proteins were selected as instruments. The main assumptions of MR are outlined in Fig. 1B. These assumptions include the relevance assumption, which suggests that the associations of genetic instruments mimicking lipid-lowering therapies were used as proxies for the pharmacological modulation of drug target genes. Another assumption is the independence assumption, which posits that genetic variants are not associated with confounding factors. Lastly, the exclusion restriction assumption states that genetic variants influence human lifespan through pathways other than the ones being studied [24]. A two-step MR analysis [25] was then performed to estimate the mediation effects of lipid-lowering variants on human longevity.

**Table 1:** Summary of genetically proxied lipid-lowering drug targets

Drug Effect	Drug Class	Drug Target	Encoding Genes	Gene Location (GRCh37 from Ensembl)	Drug Substance	Eligible IVs
↓LDL-C	Key Modulater	LDL Receptor	*LDLR*	CHR:19:11,200,038-11,244,492	–	yes
	HMGCR inhibitors	HMG-CoA reductase	*HMGCR*	CHR:5:74,632,154-74,657,929	Atorvastatin Rosuvastatin etc.	yes
	ACLY inhibitors	ATP-citrate synthase	*ACLY*	CHR:17:40,023,161-40,086,795	Bempedoic acid	no
	PCSK9 inhibitors	Proprotein Convertase Subtilisin/Kexin Type 9	*PCSK9*	CHR:1:55,505,221-55,530,525	Evolocumab Alirocumab	yes
	TC absorption inhibitors	Niemann-Pick C1-like 1	*NPC1L1*	CHR:7:44,552,134-44,580,914	Ezetimibe	yes
	ASO targeting ApoB mRNA	Apo B100	*APOB*	CHR:2:21,224,301-21,266,945	Mipomersen	yes
	ASO targeting CETP mRNA	Cholesteryl Ester Transfer Protein	*CETP*	CHR:16:56,995,762-57,017,757	Torcetrapib	yes
	BA sequestrants	Bile acids	*–*	–	Cholestyramine Colestipol	no
↓TG	Key Modulater	Lipoprotein Lipase	*LPL*	CHR:8:19,759,228-19,824,769	–	yes
	Fibrates	Peroxisome Proliferator-Activated Receptor-alpha	*PPARA*	CHR:22:46,546,424-46,639,653	Fenofibrate Gemfibrozil	no
	ANGPTL3 inhibitors	Angiopoietin-related protein 3	*ANGPTL3*	CHR:1:63,063,158-63,071,830	Evinacumab	yes
	ASO targeting ApoC-III mRNA	Apo C-III	*APOC3*	CHR:11:116,700,422-116,703,788	Volanesorsen	yes

Abbreviations: *LDL-C* Low-Density Lipoprotein Cholesterol, *TG* Total triglyceride, *LDLR* Low-Density Lipoprotein Receptor, *HMGCR* 3-hydroxy-3-methylglutaryl coenzyme A reductase, *PCKS9* Proprotein Convertase Subtilisin/Kexin Type 9, *NPC1L1* Niemann-Pick C1-like 1, *APOB* Apoprotein B-100, *CETP* Cholesteryl Ester Transfer Protein, *LPL* Lipoprotein Lipase, *ANGPTL3* Angiopoietin-related protein 3, *APOC3* Apoprotein C-III

Third, to enhance the credibility of the causal effects of drug target genes on longevity, this study also used the expression quantitative trait loci (eQTLs) summary-level data in which target genes were highly expressed as the validation method. Statistical colocalization analysis [26] was performed to calculate the probability of lipid-lowering genetic variants and lifespan phenotypes sharing common causal SNPs.

All the utilized data sources in this investigation were derived from openly accessible GWAS summary data of European populations, and detailed information is presented in Table S2. This study utilized publicly-available statistics for analysis and did not require ethical approval.

### Selection of genetic variants

For two-sample MR, single-nucleotide polymorphisms (SNP) associated with circulating lipids were identified, including LDL-C, TC, and TG, with *P ≤ 5 × 10*^*−8*^ and a linkage disequilibrium (LD) threshold (*r*^*2*^ *< 0.001)* within a clumping window of 10,000 kb distance. SNPs with inconsistent alleles in exposure and outcome GWAS databases were strictly excluded. Steiger filtering [27] was also used to identify the bidirectional effects, and variants with reverse causal effects were removed accordingly.

In drug-target MR, information on drug targets and their corresponding encoding genes were extracted from the DrugBank (https://go.drugbank.com/) and the NCBI Gene Database (https://www.ncbi.nlm.nih.gov/gene/). Eleven target genes were identified, including *LDLR*, *HMGCR*, Proprotein Convertase Subtilisin/Kexin Type 9 (*PCKS9*), ATP-citrate synthase (*ACLY*), Niemann-Pick C1-like 1 (*NPC1L1*), Cholesteryl Ester Transfer Protein (*CETP*), Apoprotein B-100 (*APOB*), *LPL*, Angiopoietin-related protein 3 (*ANGPTL3*), Apoprotein C-III (*APOC3*) and Peroxisome Proliferator Activated Receptor alpha (*PPARA*). Genetic variants within these genes that encode protein targets of lipid-lowering drugs (cis-variants) were extracted from GWAS summary data from the Global Lipids Genetics Consortium [28]. The serum levels of LDL-C and TG were used as proxies for LDL-C-lowering and TG-lowering targets, respectively. Drug target SNPs, clumped to an LD threshold of *r*^*2*^ *< 0.3 *were identified (*P ≤ 5 × 10*^*−8*^) within ±100 kb regions of the corresponding genes. *PPARA* and *ACLY* were excluded from further analysis due to insufficient numbers of SNPs identified as drug proxies.

In the validation test, available eQTLs for these drug target genes were obtained as the proxy of exposure. The eQTL summary data were extracted from the eQTL gene database (https://www.eqtlgen.org/) [29] and the Genotype-Tissue Expression (GTEx) project V8 (https://gtexportal.org/home/datasets) [30]. Common cis-eQTLs were identified at mRNA expression levels in the serum, liver or fatty tissues of lipid-lowering target genes (*FDR < 0.05*). Eligible cis-eQTLs were located within ±1 Mb of the encoded genes and had a minor allele frequency greater than 0.01 with an LD threshold of *r*^*2*^ *< 0.3*.

### Statistical analysis

The inverse-variance weighting (IVW) [31] was primarily applied to evaluate the causal effects of circulating lipids, lipid-related traits, and genetically-proxied lipid-lowering therapies on human longevity. This approach estimates the causality of a 1 standard deviation increase in exposure to genetic predictors of outcome. Beta estimates were utilized to evaluate human lifespan in the discovery data, while in the validation dataset, odds ratios were used to estimate the 90th percentile of human longevity. As we used multiple GWAS databases from large consortia, the association estimates of the same trait were combined using a meta-analysis of the fixed or random-effects model based on the heterogeneity [32].

To test the MR assumptions in the study design, the initial step involved computing the *F* statistic for each instrument. The formula *F = R*^*2*^*(n − 1 − k)/(1 − R*^*2*^*)k* was used to determine this statistic, where *R*^*2*^ represents the proportion of explained variation, *k* denotes the count of eligible SNPs, and *n* refers to the sample size [33]. Weak instrumental bias was characterized by SNPs with *F*-statistics less than 10. Statistical power was estimated using the mRnd website (https://shiny.cnsgenomics.com/mRnd/). To validate the results from the IVW method, Sensitivity tests were conducted using MR–Egger regression, maximum likelihood, weighted mode, and weighted median methods. Heterogeneity and horizontal pleiotropy were performed using the MR Egger’s intercept test and Cochran Q test. Additionally, ‘leave-one-out’ analysis was used to identify heterogeneous SNPs. To further test the validity of the drug target instruments, a positive control analysis was conducted, given the recognized benefits of lipid-lowering drugs for major coronary events and diabetes. Considering the *LD* correlation between genetic variants, the interactive heatmap matrix of pairwise linkage disequilibrium statistics was generated using the LDmatrix tool (https://ldlink.nih.gov/). Based on the results of the LD correlation matrix, LD thresholds were adjusted using narrower values (*r*^*2*^ *< 0.1* and *r*^*2*^ *< 0.01*) as additional sensitivity tests. Moreover, a Bayesian colocalization analysis [26] was performed to investigate the possibility of genetic confounding of the drug target by calculating the probability (PP.H4) of common causal SNPs between lipid-lowering genetic variants and lifespan phenotypes. Drug targets were identified with strong colocalization with lifespan as those with PP.H4 values greater than 0.85. *P values* were further adjusted from multiple testing using the false discovery rate (*FDR*) with the Benjamin-Hochberg method.

All analyses were performed in R software (4.1.0) using TwoSampleMR (github.com/MRCIEU/TwoSampleMR), MendelianRandomization, and coloc R packages. Forest plots were derived from the ggplot and ggplot2 R packages, and heatmaps were derived from the pheatmap R packages [31].

## Results

### Effects of genetic variation in circulating lipids on human lifespan

As the primary outcome, genetic traits of human longevity were estimated using both discovery and validation GWAS data. The discovery dataset contains the largest-scale GWAS summary data as a quantitative trait among 500,193 European individuals [7]. The validation GWAS data follow a binary design that comprises 36,745 Europeans, including 11,262 individuals of European ancestry who survived past the 90th survival percentile and 25,483 control individuals [21].

**Fig. 2:**
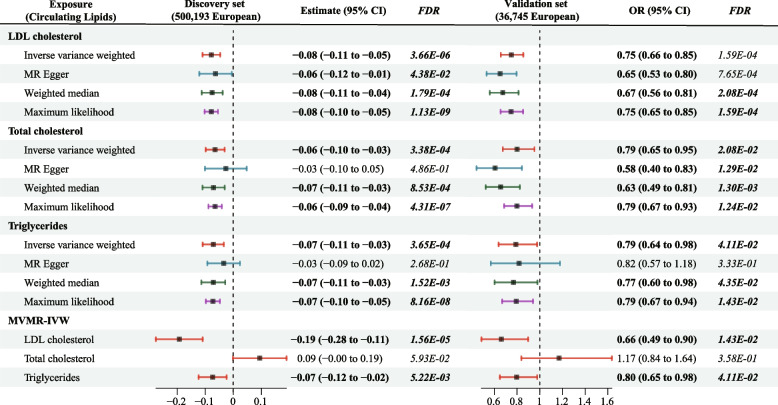
Forest plot visualizing the causal effects of circulating lipids with human lifespan. The Forest plot presented the MR estimates of circulating lipids on human lifespans in the discovery (the left plot) and validation (the right plot) datasets. Beta and 95% CI were used to estimate the effects of a 1 mmol/L change of circulating lipids (LDL cholesterol 38.67 mg/dl, Triglycerides 88.57 mg/dl) on lifespan in the discovery data (quantitative traits), whereas OR and 95% CI indicated the effect estimates of a 1 mmol/L change of circulating lipids on human longevity in the validation data (binary outcomes). Abbreviations: MVMR, Multivariable Mendelian randomization; OR: Odds Ratio; FDR, False discovery rate

After Steiger filtration and harmonizing with the discovery data, a total of 65, 75, and 51 eligible SNPs were finally revealed to be associated with LDL-C, TC, and TG, respectively. No potential weak instrument bias was indicated as the *F*-statistics for all these SNPs exceeded 10. Details of SNPs associated with the three circulating lipids are provided in Table S3-4. The univariate MR analysis showed that increases in genetically proxied LDL-C, TC, and TG were significantly associated with reduced life expectancy (all *FDR < 0.05*) in both discovery and validation datasets. The causal effect estimates of the three circulation lipids on lifespan were still significant in most sensitivity tests. Figure 2 presents the effects of genetic variation in circulating lipids on lifespan in both discovery and validation datasets. Considering potential associations that may exist among various circulating lipids, all these lipids were incorporated to estimate the direct effects of different lipid traits on the human lifespan. In multivariable MR analysis, LDL-C and triglycerides presented direct effect estimates with a reduced lifespan (all *FDR < 0.05*), whereas the adjusted effect of cholesterol on lifespan was nonsignificant. The adjusted estimate of lifespan for a 1-SD higher LDL-C was beta − 0.19 (95% CI: − 0.28 to − 0.11; *FDR = 1.56 × 10*^*−5*^) in the discovery set and OR 0.66 (95% CI: 0.49 to 0.90; *FDR = 0.013*) in the validation set. For triglycerides, it was beta − 0.07 (95% CI: − 0.12 to − 0.02; *FDR = 5.22 × 10*^*−5*^) in the discovery dataset and OR 0.80 (95% CI: 0.65 to 0.98; *FDR = 0.041*) in the validation dataset. The overall effects of genetic variation in circulating lipids on lifespan in both univariable and multivariable MR analyses are presented in Table S5.

Furthermore, the study also investigated the causal effects of dyslipidaemia-associated traits on the human lifespan (Fig. S1). Multiple data sources were used to investigate the causal effects and then combined all results to ensure adequate statistical power and the robustness of the findings. Genetic liability to BMI was associated with reduced lifespan in most GWAS consortia. For a 1-SD increase in BMI, the combined beta estimates from all GWAS consortia were beta − 0.22 (95% CI,− 0.37 to − 0.07; *P = 1.41 × 10*^*−6*^) for the lifespan in the discovery set and OR 0.92 (95% CI, 0.87 to 0.97; *P = 0.001*) in the validation set. There were also trends of causal effects in which waist circumference or the WHR was associated with decreased lifespan in the discovery set (combined estimate: beta − 0.28 (− 0.38 to − 0.18; *P = 1.96 × 10*^*−6*^). These combined results further supported that lipid-related phenotypes, including BMI and WHR, can potentially impact human lifespan. The serum levels of fasting glucose and insulin were negatively associated with lifespan, whereas no causal effects of HOMA-IR and HbA1c on lifespan were detected.

### Lipid-lowering drug targets and human longevity

**Fig. 3:**
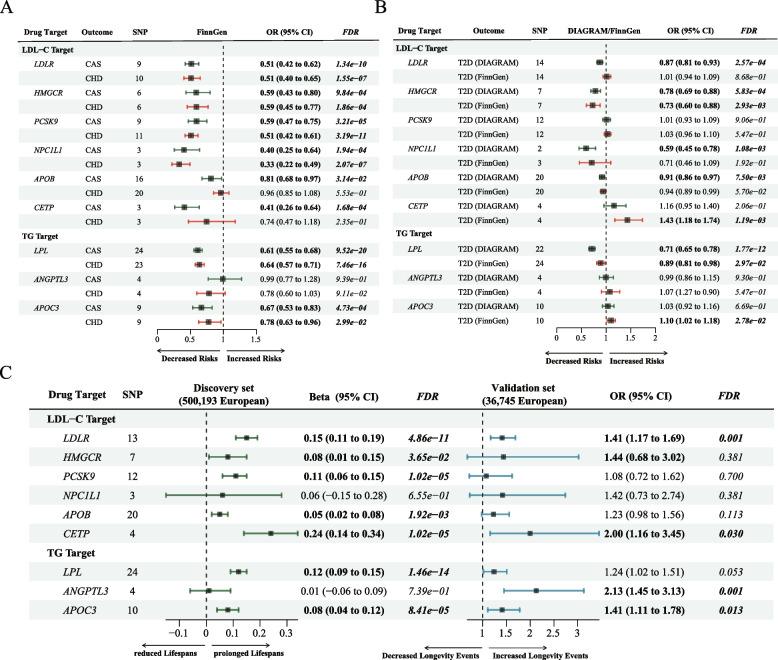
Causal effects of the genetically proxied lipid-lowering drug targets on human lifespan. **a**-**b** the IVW Results of positive control analysis were visualized in A (Major CHD events and Coronary atherosclerosis using GWAS databases from the FinnGen consortium) and B (T2D using GWAS databases from the FinnGen and DIAGRAM consortium). **c** The forest plot showed the estimated effects of 1 mmol/L lower LDL-C or TG concentration by target-specific variants in each drug target gene on human lifespan in the discovery (left) and validation sets (right), using the IVW method. Beta and 95% CI were used in quantitative outcomes, whereas OR and 95% CI were used in binary outcomes

Figure 3 presents the causal effects of 9 gene targets on the human lifespan. For the primary analysis of the discovery data, 13 variants were selected to proxy LDL lowering through *LDLR* modulator, 7 were selected for *HMGCR,* 12 were selected for *PCKS9*, 3 were selected for *NPC1L1*, 21 were selected for *LPL*, 18 were selected for *APOB*, 4 were selected for *CETP*, 4 were selected for *ANGPTL3*, and 10 were selected for *APOC3,* with all *F*-statistics exceeding 10 (Table S6-7).

In the positive control analysis, significant associations of most lipid-lowering gene targets were identified with a decreased risk of coronary atherosclerosis and major coronary heart disease (CHD) events, except for *APOB*, *CETP* (only associated with reduced coronary atherosclerosis risks), and *ANGPTL3* (not associated with either CHD or coronary atherosclerosis) (Fig. 3A). For the T2D outcome, LDL-C-lowering *HMGCR* and TG-lowering *LPL* variants had causal effects on reduced T2D risks in both FinnGen and DIAGRAM databases (Fig. 3B).

The LDL-C-lowering *LDLR* variants were associated with increased lifespan (IVW: beta 0.15; 95% CI: 0.11 to 0.19; *P = 1.08 × 10*^*−11*^*, FDR = 4.86 × 10*^*−11*^), and the positive causal effect was replicated in the validation set using the 90th percentile of longevity GWAS data (IVW: OR 1.41; 95% CI: 1.17 to 1.69; *P = 2.59 × 10*^*−4*^*, FDR = 0.001*). Genetically instrumented *CETP* and *APOC3* inhibitors were also associated with increased lifespan in both discovery and validation data. The LDL-C-lowering variants of *HMGCR* and *PCKS9,* and the triglyceride-lowering variants of *LPL* and *APOB* only presented positive effects of increased life expectancy in the discovery set rather than the validation set. No statistical evidence of association were observed between *NPC1L1* and human longevity in either dataset (Fig. 3C). Sensitivity tests showed consistent trends in the estimates, with no statistical evidence of bias from horizontal pleiotropy and heterogeneity (Table S8-9). The sensitivity test with different LD thresholds is presented in Fig. S2 and Table S10. The statistical power is presented in Table S11.

**Fig. 4:**
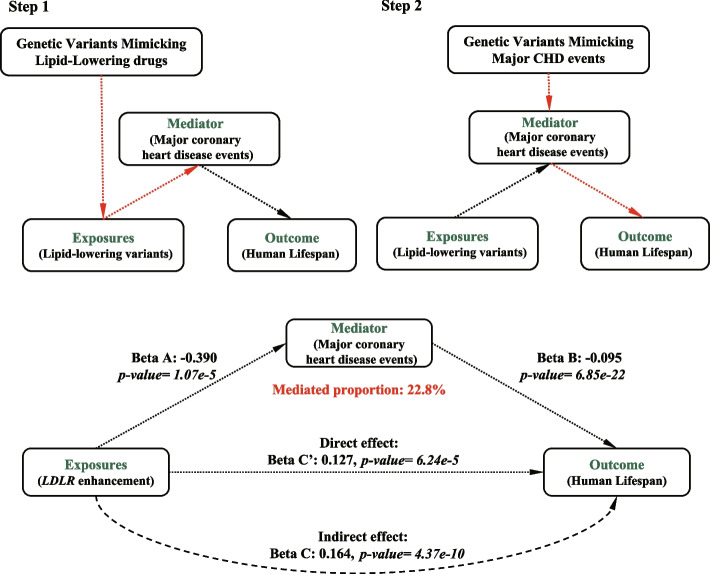
Flowchart of the two-step Mendelian randomization for mediation analysis. Two-step Mendelian randomization, where the effect of genetically proxied lipid-lowering target gene variants (Beta A) on the mediator (Major CHD events) and mediator on the human lifespans (Beta B) are estimated separately, using separate genetic instrumental variables for both the exposure and mediator. These estimates are then multiplied together to estimate the indirect effect of the mediator (Beta A*Beta B). Beta C represents the total effect of the exposure to the outcome. The indirect effect (Beta C′) of the exposure to the outcome was estimated using the format: Beta C-Beta A*Beta B. Abbreviations: *LDLR*, Low-Density Lipoprotein Receptor; CHD, Major coronary heart disease; OR: Odds Ratio; FDR, False discovery rate

To further investigate the potential links between lipid-lowering drug targets and human longevity, the role of the major CHD event was examined as a possible mediator (Fig. 4A). Major CHD events may be causally related to both the effect of lipid-lowering *LDLR* variants on CHD (step one) and the effect of CHD on lifespan (step two). After adjusting for CHD, effect of *LDLR* on lifespan reduced from 0.164 (indirect effect, 95% CI 0.112 to 0.215, *P = 4.37 × 10*^*−10*^) to 0.127 (direct effect, 95% CI, 0.093 to 0.159, *P = 6.24 × 10*^*−5*^), indicating that major CHD events play a significant role as a mediator, accounting for 22.8% of the mediation effect for triggering the lipid-lowering effects of *LDLR* on extended human lifespan (Fig. 4B). Using similar two-step mediation MR analyses, the major CHD event was also identified as the mediators of *PCSK9* (5.1%) and *APOC3* (26.1%) for triggering the lipid-lowering effects that extended human lifespan. Coronary atherosclerosis plays a role as a mediator, accounting for 37.0% of the mediation effect for triggering the lipid-lowering effects of *CETP* on extended human lifespan. T2D, obesity, or other lipid-associated traits had no mediation effects on the associations of *LDLR, PCSK9, CETP, and APOC3* variants with lifespan.

### Validating the effects of drug target genes on lifespan in genotype tissues

**Fig. 5:**
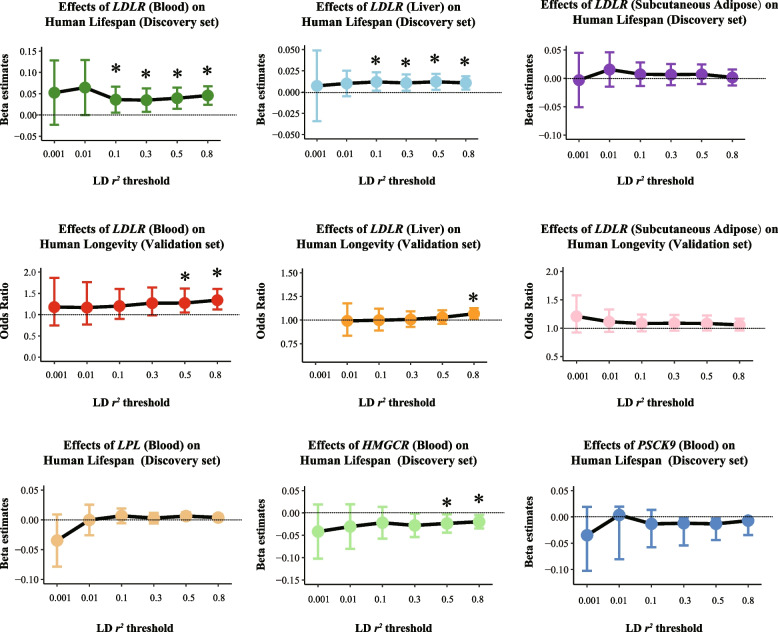
Associations of target genes expressed in tissues with lifespan under increasingly liberal LD-clumping thresholds. The graph displays the R2 values below the x-axis. The IVW method was applied as the main analysis approach. Beta and 95% CI were used in quantitative outcomes, whereas OR and 95% CI were used in binary outcomes. *, **, *** stands for *p*-value < 0.01, 0.005 and 0.001, respectively

As the lipid-lowering variants of *LDLR, CETP,* and *APOC3* showed robust statistical evidence of the association with longevity in both discovery and validation datasets, the eQTL data of the three genes highly expressed in blood samples, liver, and subcutaneous adipose tissues were used as instruments. We observed that a 1-SD increase in *LDLR* expression was associated with increased lifespan in both blood tissue (beta 0.03; 95% CI: 0.01 to 0.04; *P = 0.012*) and liver tissue (beta 0.01; 95% CI: 0.001 to 0.02; *P = 0.038*). For *CETP,* and *APOC3,* there was no statistical evidence of any genetic association with longevity in the blood or subcutaneous adipose tissues. (Fig. 5, Table S12).

As the lipid-lowering variants of *HMGCR, PCKS9, LPL*, and *APOB* only presented positive effects of increased life expectancy in the discovery set, we also validated these variants in the blood tissues. The higher expression of *HMGCR* was causally associated with reduced lifespan (beta − 0.028; 95% CI: − 0.050 to − 0.002; *P = 0.037*), whereas there was little statistical evidence of any genetic association with longevity in the serum expression of *PCKS9, LPL*, and *APOB.* Additional sensitivity analyses on LD thresholds using narrower values (*r*^*2*^ *< 0.1*, *r*^*2*^ *< 0.01*, and *r*^*2*^ *< 0.001*) showed no significant effect after widening the confidence intervals.

Next, the colocalization analysis was applied to verify whether the *LDLR* expressed in relevant tissues had common causal SNPs in common with the lifespan phenotype. The probability of the causal variants between *LDLR* and lifespan was 1.18% in PP.H4 in the discovery dataset and 2.14% in the validation dataset (Fig. S3, Table S13).

**Fig. 6:**
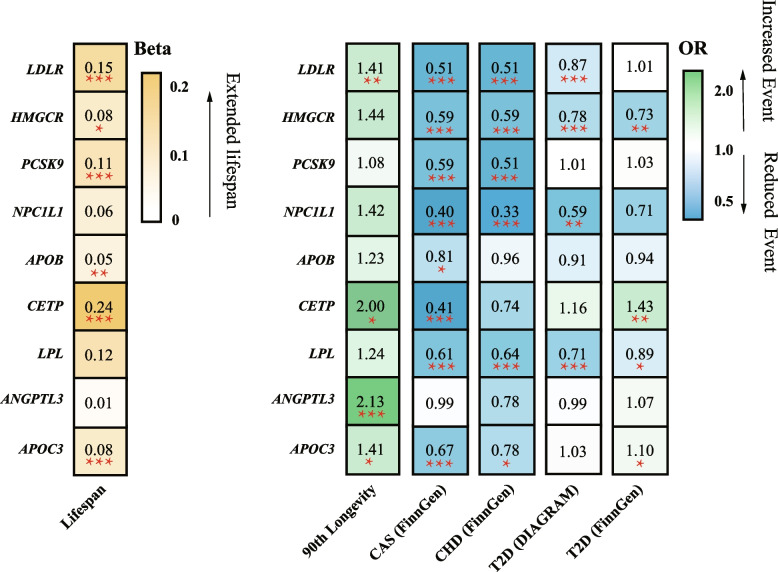
Summary of the study. Heatmap visualization of the Beta or OR estimates of lipid-lowering drug targets on human lifespan, cardiovascular diseases, and T2D. The figure displays a matrix with rows representing gene targets of lipid-lowering agents and columns representing outcomes from different GWAS consortiums. The values in each square indicate the Beta or OR estimates and are color-coded based on their specific values. The left heatmap applied the gradually deepening yellow, indicating the increasing Beta values. The right heatmap applied the deepening green indicating the increasing OR values and the deepening blue representing the decreasing ORs

The main results of the study are presented in Fig. 6.

## Discussion

This study revealed that circulating lipid LDL-C and TG had causal effects on human life expectancy, and lipid-lowering interventions targeting *LDLR* and some new targets, such as *PCSK9, CETP,* and *APOC3,* may play a causal role in prolonging lifespan. The mediation analysis further suggested that genetic mimicry of these genes may extend the human lifespan by reducing the risks of major CHD events.

Among the nine lipid-lowering drug gene targets, the *LDLR* gene may be the most promising drug target that is causally associated with human longevity. In a large-scale longevity-associated GWAS, *Timmers* et al. [7] reported that *LDLR* may be a potential gene target for extending human lifespan. Although very few studies have directly investigated the relationship between the *LDLR* gene and human longevity, previous evidence suggested that the *LDLR* gene plays a beneficial role in maintaining physiological homeostasis. For instance, *LDLR* knockout mice exhibited reduced oligodendrocyte activity [34] and a shorter lifespan of osteoclasts in vitro [35]. Additionally, another animal study demonstrated that *LDLR* deficiency led to increased neurological deficits and long-term cognitive impairment [36]. Hence, the *LDLR* gene may be involved in regulating physiological homeostasis in human tissues, thereby indirectly impacting human longevity. Another possible explanation is that *LDLR* is also an indirect gene target for statin therapies because interfering with hepatic cholesterol synthesis could compensate for the increase in the de novo synthesis of *LDLR* and then transport more *LDLR* to the hepatocellular membranes [37]. A few randomized trials and meta-analyses have already demonstrated that statin use is associated with extended lifespan by reducing the mortality rate associated with atherosclerotic cardiovascular events [12, 13, 38]. Since the main pharmacological target for statins is the *HMGCR* gene, this study revealed that genetic mimics of *HMGCR* inhibitors were also associated with prolonged lifespan. However, the causal effects of *HMGCR* on lifespan were not further identified in the eQTL database. These results suggest that the extended lifespan caused by statins might be achieved through upregulating *LDLR* more than *HMGCR*, as *LDLR* showed more convincing genetic evidence of causally affecting human longevity than *HMGCR*. In addition, the mediation analysis further suggested that genetic mimicry of *LDLR* enhancement may extend the human lifespan by reducing the risks of major CHD events, which is also consistent with the results from clinical trials.

In addition to the *LDLR* gene, several nonstatin gene targets also showed genetic associations with human lifespan. These genes include *PCSK9, CETP,* and *APOC3.* Currently, *PCSK9* targeting drugs are newer nonstatin drugs, including intracellular PCSK9 inhibitors (inclisiran) and extracellular *PCSK9* inhibitors (*PCSK9* mAbs) [22]. Inclisiran was approved in December 2021 as a supplement for maximally tolerated statin use in adult patients with cardiovascular disorders who require additional lipid-lowering therapies [22]. The effect of inclisiran on cardiac-related mortality or morbidity has not been established. Two clinical trials are currently underway [22, 39]. Therefore, this study also provides genetic evidence for these clinical trials that *PCSK9* inhibitors may have promising effects on extending lifespan. Another newer nonstatin agent identified in this study as being able to prolong lifespan is the *CETP* inhibitor. Evidence from large observational studies suggests that genetic *CETP* deficiency not only increases HDL-C but also lowers LDL-C. Therefore, low *CETP* activity is associated with high risks of cardiovascular disease [40]. *CETP* inhibitors have been a topic of controversy due to the termination of several previous clinical trials involving torcetrapib or dalcetrapib [41]. These trials were halted either because of increased risks of major CHD events [42] or lack of efficacy [43]. However, recent MR studies have confirmed that *CETP*, similar to *HMGCR, PCSK9*, and *NPC1L1* genes, can reduce LDL-C levels [8]. As a result, several new drugs are being developed. Anacetrapib, the most recent *CETP* inhibitor, has shown effectiveness in reducing major CHD events [44]. Currently, obicetrapib is the newest and the most potent *CETP *inhibitor, achieving up to 45 and 34% reductions in LDL-C and apoB, respectively [45]. The present research, in comparison to previous MR studies, confirms that *CETP *causally reduces the risk of CAS events and has a long-term impact on extending human lifespan. This provides further genetic evidence for developing these novel nonstatin drugs in the future.

Furthermore, *APOC3* was also a potential nonstatin target associated with elongated lifespan. *APOC3 *is a multilamellar apolipoprotein that affects TG catabolism [46]. The overexpression of ApoC-III can lead to unnecessary accumulation of TG in blood vessels and cause hypertriglyceridaemia. A meta-analysis including 12 eligible epidemiological studies supported a strong correlation of apoC-III with cardiovascular risks [47]. Therefore, it has been found as an independent risk factor for cardiovascular disease. Volanesorsen is a chemically modified anti-*APOC3* antisense oligonucleotide [48]. To date, randomized trials of Volanesorsen on treating severe hypertriglyceridaemia are underway, and the prospect looks hopeful [48]. The encouraging results of this study demonstrate that genetic inhibition of *APOC3* is causally associated with increased life expectancy and longevity events and provide genetic evidence for the development of this novel therapy.

Drug-target MR is a potential approach to discover innovative preventive therapies and treatments, particularly for longevity aspects where conducting clinical trials is frequently impractical. However, all findings in this study require careful interpretation. First, as there is no established standard for determining the LD threshold used for drug-target MR, 0.3 was used as the main LD threshold in this study. To evaluate the robustness of results, sensitivity tests were performed using different LD thresholds. When *r*^*2*^ was set to less than 0.1, *LDLR*, *PCSK9*, *APOB*, *LPL*, and *APOC3* still showed positive results in the discovery dataset. However, when a more stringent LD value (*r*^*2*^ *< 0.01*) was applied, only *PSCK9*, *LPL*, and *APOC3* remained significant. Therefore, the choice of different LD values might impact the reliability of the causal relationship between certain genes (such as *LDLR* and *CETP*) and human longevity, and high *r*^*2*^ values could sometimes indicate overfitting or may reflect pleiotropic effects, which in turn might lead to biased estimates. Second, MR based on eQTL data combined with colocalization is a recently developed method for identifying causal relationships between drugs and diseases [49]. The eQTL-based MR analyses may yield similar results compared to traditional biomarker MR. Therefore, eQTL data was used as validation for conventional drug-target MR. However, it is important to note that the sample size of eQTL data may be relatively small, resulting in weaker evidence compared to the results from drug-target MR. Thus, these results still need further validation using larger-scale GWAS data or pQTL databases. Third, we found inconsistent results of statin use on T2D during drug-target MR analyses from previous epidemiological studies. Additionally, a significant association between *ANGPTL3* and major CHD events was not observed. These inconsistencies may be because multiple genetic proxies of lipid-lowering drugs were employed, and the observed causal effects on lifespan may be attributed to genetically proxied drug target genes rather than the whole impact of specific medications in the real world. Although drug-target MR analysis can provide the direction of causal effects, it cannot accurately determine the magnitude of clinical benefits. Therefore, additional high-quality randomized trials or large-scale epidemiological studies are necessary to further validate these findings.

## Strength and limitation

This study was the first to utilize drug-target MR as an innovative approach to uncover the genetic association between nine lipid-lowering drug-target genes and human longevity. These genes include commonly-used statins as well as newer nonsatain gene targets, allowing for the investigation of potential benefits associated with these novel nonsatain drugs. However, the study also has several limitations. Despite utilizing the largest binary longevity GWAS data, the sample size in the validation set remains relatively small, and several results lacked sufficient statistical power. In addition, the investigation of the causal impacts of bempedoic acid or fibrates on human lifespan is unattainable owing to the insufficient numbers of target-specific SNPs in the Global Lipids Genetics Consortium. Last, despite the availability of GWAS data for Asian populations, a lack of alignment were observed between the significant genetic variants identified in the Asian population and those identified in this study. Therefore, these findings are limited to individuals of European ancestry and require confirmation in other populations. The availability of more larger-scale GWAS data in Asian populations may facilitate further analysis.

## Conclusions

In summary, this study provides genetic evidence that statins and newer nonstatin therapies, especially acting through the *LDLR* pathway, have potential causal effects on reducing cardiovascular risks and prolonging human longevity. Lipid-related gene targets, such as *PCSK9, CETP*, and *APOC*3, might potentially regulate human lifespan, thus offering promising prospects for developing newer nonstatin therapies. Additionally, this study emphasizes the potential benefits of lipid-lowering interventions in preventing cardiovascular disease and extending lifespan.

### Supplementary Information

**Additional file 1:** **Table S1.** The STROBE statement guideline checklist of this study.

**Additional file 2:** **Table S2.** Date resources of the exposures and outcomes used in this study. **Table S3.** The instrumental variables used for circulating lipids in the discovery datasets. **Table S4.** The instrumental variables used for circulating lipids in the validation datasets. **Table S5.** Association of circulating lipids with human Lifespan in the discovery and validation datasets. **Table S6.** Genetically instrumented lipid-lowering genetics variants of target genes in discovery datasets. **Table S7.** Genetically instrumented lipid-lowering genetics variants of target genes in validation datasets. **Table S8.** Effects of genetically proxied lipid-lowering variants on Human lifespan. **Table S9.** Results of heterogeneity and pleiotropy tests for the causal effects of circulating Lipids on human lifespan. **Table S10.** Sensitivity analysis using different LD *r*^*2*^ thresholds of the Lipid-lowering gene variants. **Table S11.** Statistical power for drug-target MR analyses. **Table S12.** Genetic association between the expression of lipid-lowering drug target genes in tissues and human lifespan. **Table S13.** Colocalization analysis for the serum *LDLR* expression on Human Lifespan.

**Additional file 3:** **Fig. S1.** Forest plot visualizing the causal effects of lipid-associated traits with human lifespan. The Forest plot presented the MR estimates of circulating lipids on human lifespans in the discovery (the left plot) and validation (the right plot) datasets. Beta estimates and 95%CI were utilized to evaluate human lifespan in the discovery data, while in the validation dataset, Odds Ratio and 95%CI were used to estimate the 90th human longevity. As we used multiple GWAS databases from large consortia, the association estimates of the same trait were combined using a meta-analysis of the fixed or random effects model based on the heterogeneity. Abbreviations: OR: Odds Ratio; BMI, Body mass index; WHR, Waist-to-hip ratio; FBG, Fasting blood glucose;FI, Fasting Insulin; HOMO-IR, Homeostatic model assessment of insulin resistance.

**Additional file 4:** **Fig. S2.** Triangular linkage disequilibrium (LD) matrix plot of SNP markers of each lipid-lowering drug target gene. The plots were produced using pairwise r2 estimates of LD using the LD LDmatrix Tool (https://ldlink.nih.gov/). The pairwise r2 values are given inside the boxes. Combinations with significant LD between the same types of gene alleles were marked with *. *, **, *** stands for *p*-value < 0.01, 0.005 and 0.001, respectively.

**Additional file 5:** **Fig. S3.** Visualization of Colocalisation analysis. Colocalisation analysis of the cis-eQTL for the LDLR expressed in blood tissues and human lifespan in the discovery (A) and validation (B) datasets. Each dot represents an SNP at the LDLR locus. The dots in the scatter plots are colored according to their linkage disequilibrium to the colocalisation lead variant. The *P* values of the SNPs in the LDLR locus were extracted from the eQTL-Gen database in the blood tissue and from the human lifespan GWAS databases.

## Data Availability

All data generated or analysed during this study are included in this published article and its supplementary information files. The availability of all the data used in the study was summarized in Table S2.

## Abbreviations

GWAS: Genome-wide association studies
LDLR: Low-Density Lipoprotein Receptor
LPL: Lipoprotein Lipase
LDL-C: Low-Density Lipoprotein Cholesterol
TC: Total cholesterol
TG: Total triglyceride
HMGCR: 3-hydroxy-3-methylglutaryl coenzyme A reductase
MR: Mendelian randomization
STROBE-MR: Strengthening the Reporting of Observational Studies in Epidemiology using Mendelian Randomization
BMI: Body mass index
WHR: Waist-to-hip ratio
FBG: Fasting blood glucose
FI: Fasting Insulin
HOMO-IR: Homeostatic model assessment of insulin resistance
eQTLs: expression quantitative trait loci
SNPs: Single nucleotide polymorphisms
LD: Linkage disequilibrium
ACLY: ATP-citrate synthase
PCKS9: Proprotein Convertase Subtilisin/Kexin Type 9
NPC1L1: Niemann-Pick C1-like 1
APOB: Apoprotein B-100
CETP: Cholesteryl Ester Transfer Protein
ANGPTL3: Angiopoietin-related protein 3
APOC3: Apoprotein C-III
GTEx: Genotype-Tissue Expression
FDR: False discovery rate
CHD: Major coronary heart disease
T2D: Type 2 diabetes.
