# Supplementary material for: Genetic insights into the association of statin and newer nonstatin drug target genes with human longevity: a Mendelian randomization analysis
Source: Lipids Health Dis. 2023 Dec 12;22:220. doi: 10.1186/s12944-023-01983-0 (PMC10714481; doi:10.1186/s12944-023-01983-0)
Supplement: Supplementary file 3 — Additional file 3: Fig. S1. Forest plot visualizing the causal effects of lipid-associated traits with human lifespan. The Forest plot presented the MR estimates of circulating lipids on human lifespans in the discovery (the left plot) and validation (the right plot) datasets. Beta estimates and 95%CI were utilized to evaluate human lifespan in the discovery data, while in the validation dataset, Odds Ratio and 95%CI were used to estimate the 90th human longevity. As we used multiple GWAS databases from large consortia, the association estimates of the same trait were combined using a meta-analysis of the fixed or random effects model based on the heterogeneity. Abbreviations: OR: Odds Ratio; BMI, Body mass index; WHR, Waist-to-hip ratio; FBG, Fasting blood glucose;FI, Fasting Insulin; HOMO-IR, Homeostatic model assessment of insulin resistance. [file 12944_2023_1983_MOESM3_ESM.pdf]

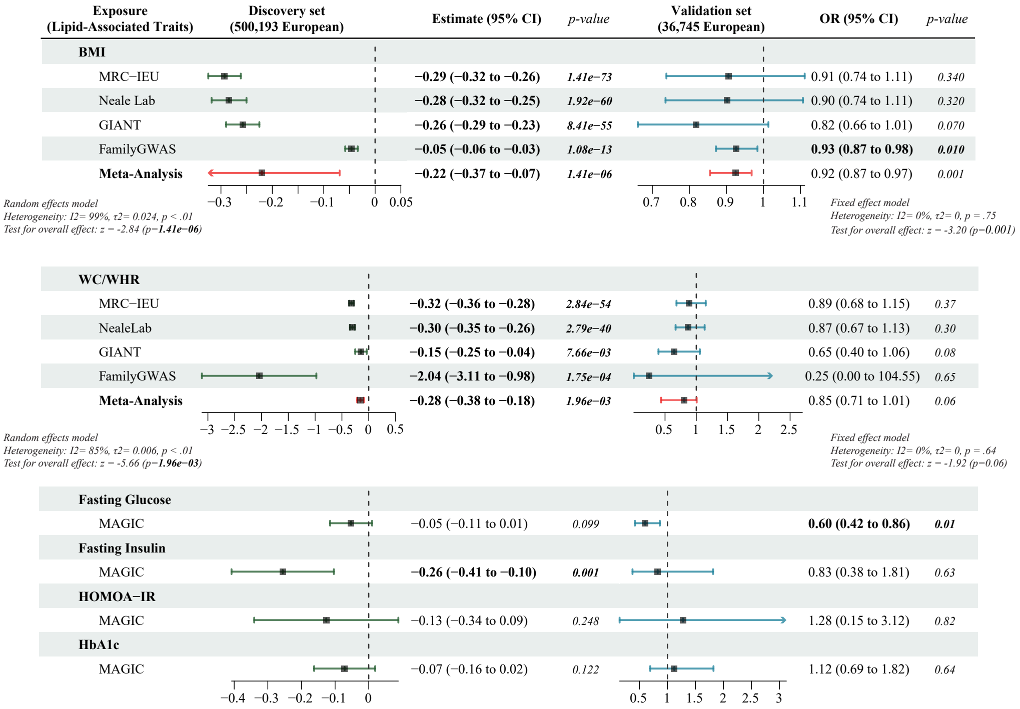

Figure S1. Forest plot visualizing the causal effects of lipid-associated traits with human lifespan. The Forest plot presented the MR estimates of circulating lipids on human lifespans in the discovery (the left plot) and validation (the right plot) datasets. Beta estimates and 95%CI were utilized to evaluate human lifespan in the discovery data, while in the validation dataset, Odds Ratio and 95%CI were used to estimate the 90th human longevity. As we used multiple GWAS databases from large consortia, the association estimates of the same trait were combined using a meta-analysis of the fixed or random effects model based on the heterogeneity. **Abbreviations:** OR: Odds Ratio; BMI, Body mass index; WHR, Waist-to-hip ratio; FBG, Fasting blood glucose;FI, Fasting Insulin; HOMO-IR, Homeostatic model assessment of insulin resistance.
