# Supplementary material for: Genetic insights into the association of statin and newer nonstatin drug target genes with human longevity: a Mendelian randomization analysis
Source: Lipids Health Dis. 2023 Dec 12;22:220. doi: 10.1186/s12944-023-01983-0 (PMC10714481; doi:10.1186/s12944-023-01983-0)
Supplement: Supplementary file 4 — Additional file 4: Fig. S2. Triangular linkage disequilibrium (LD) matrix plot of SNP markers of each lipid-lowering drug target gene. The plots were produced using pairwise r2 estimates of LD using the LD LDmatrix Tool (https://ldlink.nih.gov/). The pairwise r2 values are given inside the boxes. Combinations with significant LD between the same types of gene alleles were marked with *. *, **, *** stands for p-value < 0.01, 0.005 and 0.001, respectively. [file 12944_2023_1983_MOESM4_ESM.pdf]

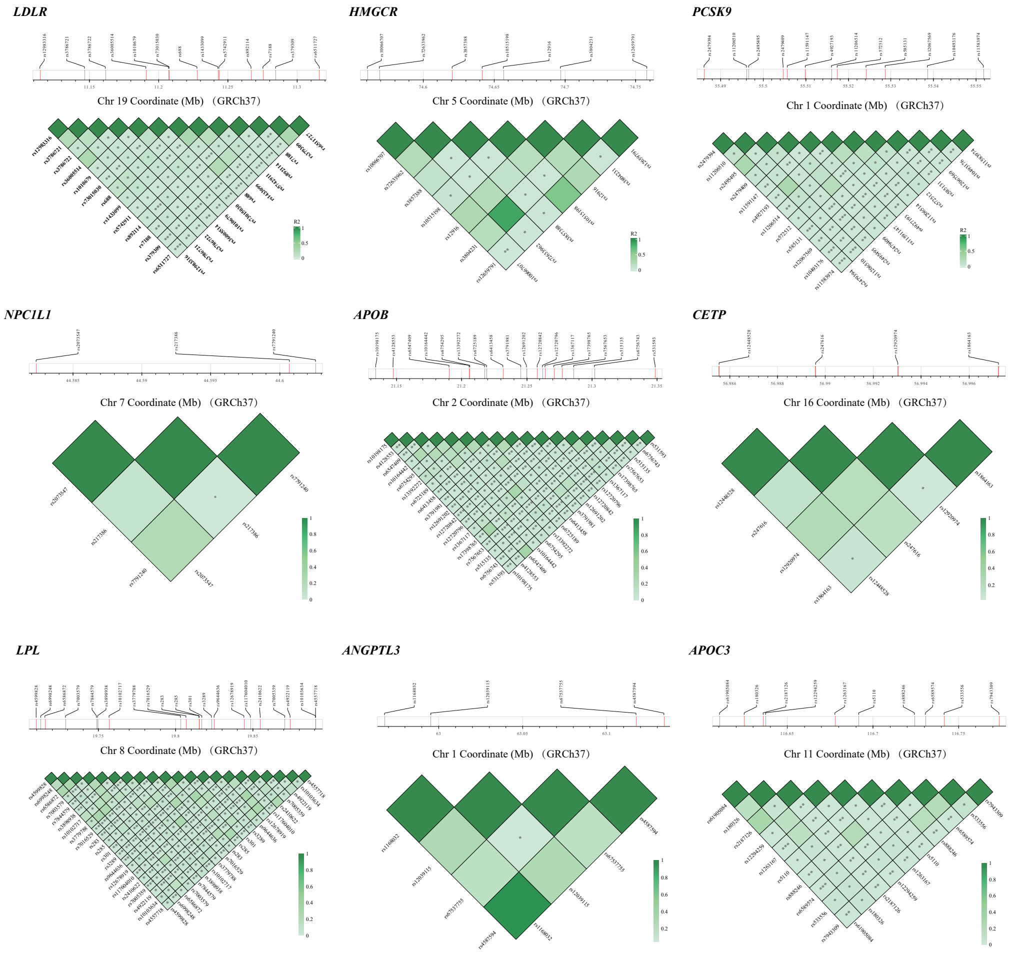

Figure S2. Triangular linkage disequilibrium (LD) matrix plot of SNP markers of each lipid-lowering drug target gene. The plots were produced using pairwise r2 estimates of LD using the LD LDmatrix Tool (https://ldlink.nih.gov/). The pairwise r2 values are given inside the boxes. Combinations with significant LD between the same types of gene alleles were marked with *. *, **, *** stands for p-value < 0.01, 0.005 and 0.001, respectively.
