# Supplementary material for: Genetic insights into the association of statin and newer nonstatin drug target genes with human longevity: a Mendelian randomization analysis
Source: Lipids Health Dis. 2023 Dec 12;22:220. doi: 10.1186/s12944-023-01983-0 (PMC10714481; doi:10.1186/s12944-023-01983-0)
Supplement: Supplementary file 5 — Additional file 5: Fig. S3. Visualization of Colocalisation analysis. Colocalisation analysis of the cis-eQTL for the LDLR expressed in blood tissues and human lifespan in the discovery (A) and validation (B) datasets. Each dot represents an SNP at the LDLR locus. The dots in the scatter plots are colored according to their linkage disequilibrium to the colocalisation lead variant. The P values of the SNPs in the LDLR locus were extracted from the eQTL-Gen database in the blood tissue and from the human lifespan GWAS databases. [file 12944_2023_1983_MOESM5_ESM.pdf]

The serum *LDLR* expression on Human Lifespan

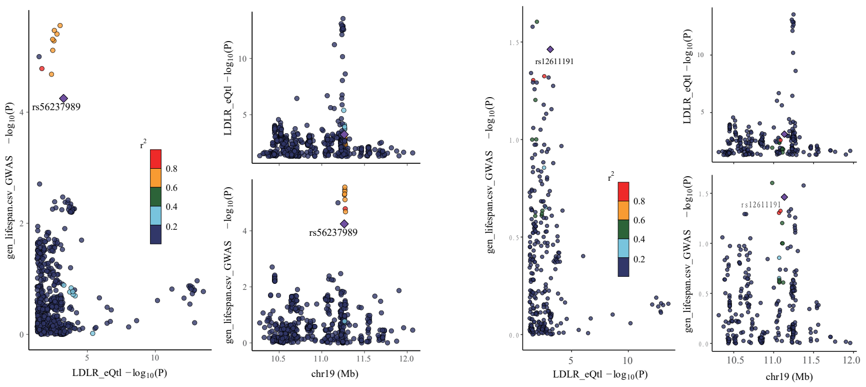

Human Lifespan (Discovery date set) **Human Lifespan (Validation date set)**

Figure S3 Visualization of Colocalisation analysis. Colocalisation analysis of the cis-eQTL for the LDLR expressed in blood tissues and human lifespan in the discovery (A) and validation (B) datasets. Each dot represents an SNP at the LDLR locus. The dots in the scatter plots are colored according to their linkage disequilibrium to the colocalisation lead variant. The P values of the SNPs in the LDLR locus were extracted from the eQTL-Gen database in the blood tissue and from the human lifespan GWAS databases.
